# Neuropathy with Cerebral Features Induced by Nitrous Oxide Abuse—A Case Report

**DOI:** 10.3390/toxics11120959

**Published:** 2023-11-25

**Authors:** Erik Lindeman, Sara Melin, Martin Paucar, Richard Ågren

**Affiliations:** 1 Swedish Poisons Information Centre, 17154 Stockholm, Sweden; erik.lindeman@gic.se; 2 Department of Clinical Neuroscience, Karolinska Institutet, 17177 Stockholm, Sweden; sara.melin@regionstockholm.se (S.M.); martin.paucar-arce@regionstockholm.se (M.P.); 3 Department of Neurology, Karolinska University Hospital, 17176 Stockholm, Sweden; 4 Department of Physiology and Pharmacology, Karolinska Institutet, 17177 Stockholm, Sweden

**Keywords:** neuropathy, nitrous oxide, athetosis, hyperkinesia, basal ganglia

## Abstract

Nitrous oxide abuse may cause functional cobalamin deficiency and subsequent damage to the peripheral nerves, the spinal cord, and the brain, a symptom complex best described by the term cobalamin neuropathy. Here, we report a case of cobalamin neuropathy with uncommon cerebral symptomatology following nitrous oxide intoxication and contextualize the symptomatology. A 22-year-old male with a history of mixed drug dependency presented at the emergency room after inhaling six 615 g cylinders, equal to ~1800 L, of nitrous oxide daily for two weeks. His main complaints were rapidly progressing paresthesias and gait difficulties, but he was also found to suffer from memory impairment and signs of extrapyramidal pathology in the form of dystonic posturing and athetosis. Neuroimaging demonstrated spinal cord hyperintensities consistent with subacute combined degeneration. The patient had low serum cobalamin and high plasma homocysteine, suggesting cobalamin neuropathy. After commencing treatment with parenteral hydroxocobalamin, plasma homocysteine normalized. The extrapyramidal symptoms disappeared during the first days of treatment, whereas the cognitive and peripheral symptoms only partially resolved over the following 20 days. This case highlights how neurological symptoms such as hyperkinetic movements and memory impairment may be associated with chronic nitrous oxide abuse. It is unclear to what extent these and other symptoms of cobalamin neuropathy are reversible, which underscores the public health concern.

## 1. Introduction

The recreational use of nitrous oxide (N_2_O) has become a part of mainstream youth culture in Europe in recent years. The sale of N_2_O is largely unregulated, despite multiple reports describing a rising incidence of severe neurological complications caused by its abuse [1]. Mechanistically, N_2_O is an anaesthetic gas of low potency (minimal alveolar concentration slightly greater than 1 atmosphere) that exerts its effect by antagonizing N-methyl-D-aspartate receptors [2,3]. Effect onset and offset are among the fastest for all the anaesthetic gases, and N_2_O is rapidly excreted unchanged through exhalation when administration is discontinued [1]. N_2_O oxidizes the cobalt atom in cobalamin (vitamin B_12_), rendering the affected vitamin inactive [4]. This reaction very rarely causes clinical symptoms in occasional N_2_O exposure, such as when the gas is used for procedural sedation or anaesthesia [5,6]. In prolonged continuous exposure, such as during sedation with N_2_O in an intensive care setting or during abuse, the gas can cause a severe functional cobalamin deficiency [7,8,9]. Although N_2_O toxicity was first reported in 1956, its metabolic underpinnings were not elucidated until the end of the 1970s, at a time when N_2_O had been in clinical use for well over 100 years [7,8,9,10,11].

The discovery of the destructive effects of N_2_O on cobalamin stimulated the development of animal models where the gas was used to cause cobalamin deficiency, a state that had previously been notoriously difficult to induce in the laboratory [12,13]. These models have, in turn, increased the understanding of the mechanisms underlying “subacute combined degeneration of the spinal cord” (SCD) [11]. It is clear, however, that cobalamin deficiency causes damage to all parts of the nervous system, including the peripheral nerves and the brain in addition to the spinal cord. The term cobalamin neuropathy has been suggested as an alternative to SCD to better describe the disease complex, and this term will be used henceforth [14,15].

Neuropathy is recognized as the most disabling complication of cobalamin deficiency since pernicious anaemia became a survivable disease after the introduction of liver extract therapy in the 1920s. This complication has been present in about 40% of patients with pernicious anaemia at the time of diagnosis for over half a century [16,17]. Until recently, N_2_O abuse as a cause of cobalamin deficiency and neuropathy has been a marginal occurrence. In 1995, there were 30 published cases, most involving dentists using their anaesthetic equipment as the source of N_2_O [15]. Twenty years later, the number of published cases were still under 100, and cases of cobalamin neuropathy in recreational N_2_O users were still presented as “neurological rarities” [18,19]. Prior to 2018, N_2_O abuse was practically unheard of at the Swedish Poisons Information Centre (PC). However, in the last couple of years, the situation has changed dramatically. In 2022, the PC was contacted from hospitals regarding 150 individual cases of complications from N_2_O use, a number already surpassed in 2023, with a majority of cases around 20 years of age and displaying symptoms consistent with cobalamin neuropathy [20,21]. Similar experiences are reported by poison centres in other countries [22]. Currently, the Swedish PC is contacted regarding more cases of cobalamin neuropathy in a single year than had been reported in the entire medical literature less than ten years ago. The entire cohort of Swedes under 30 years of age with pernicious anaemia, who have had symptoms of cobalamin neuropathy during their lifetime, can be estimated to consist of 80 patients (assuming 40% with neuropathy and adjusting for contemporary population size), less than the number of cases now caused by N_2_O in a single year in the same age group [14,23]. Furthermore, the severity of symptoms seen in many of these young people is of a magnitude that would have astounded most physicians familiar with pernicious anaemia in the post-liver-extract era. In the following sections, we present a case notable for the rapid development of severe symptoms attributed to the peripheral and central nervous system, including the basal ganglia. The case serves as a basis for a discussion of the metabolic mechanisms underlying the complications of cobalamin neuropathy, relevant for a new era where N_2_O-evoked cobalamin neuropathy is no longer a “neurological rarity”.

## 2. Case Description

A 22-year-old male was referred to the emergency room for anaemia, fatigue, balance difficulties with falls, insomnia, and impaired memory. He had a longstanding history of mixed drug use which included cannabis, tramadol, and N_2_O. Three weeks before presenting, the patient had dramatically escalated his N_2_O use to six 615 g cylinders per day. During this binge, he developed paresthesias in the hands and feet, progressive gait difficulties, and general weakness. The emergence of these symptoms caused the patient to abstain from N_2_O use for a week prior to admission. However, the neurological symptoms kept progressing. During the first days in hospital, dystonic posturing and athetosis were noticed. Cognitive evaluation using the Montreal Cognitive Assessment (MoCA) test yielded 23/30 points [24].

The patient was oriented with blunt affects but cooperative. His response latency was prolonged and his facial expression hypomimic, although there were no signs of tremor or bradykinesia. His gait was shuffling and unsteady with reduced arm swinging, and he was unable to perform tandem gait or to jump on his right leg. In addition, examination revealed mild dysmetria and nystagmus. He had distal and proximal weakness, which was more pronounced in the right leg, and muscle strength was graded 4/5 in both arms and legs. Both Romberg’s and Lhermitte’s signs were positive. Muscle tonus and reflexes were normal, and the Babinski’s sign and clonus were absent. However, there was a marked reduction in sensation and loss of proprioception in the legs, and an absence of the sense of vibration in the malleoli. The patient had urinary retention and required a catheter for the first days.

**Figure 1:**
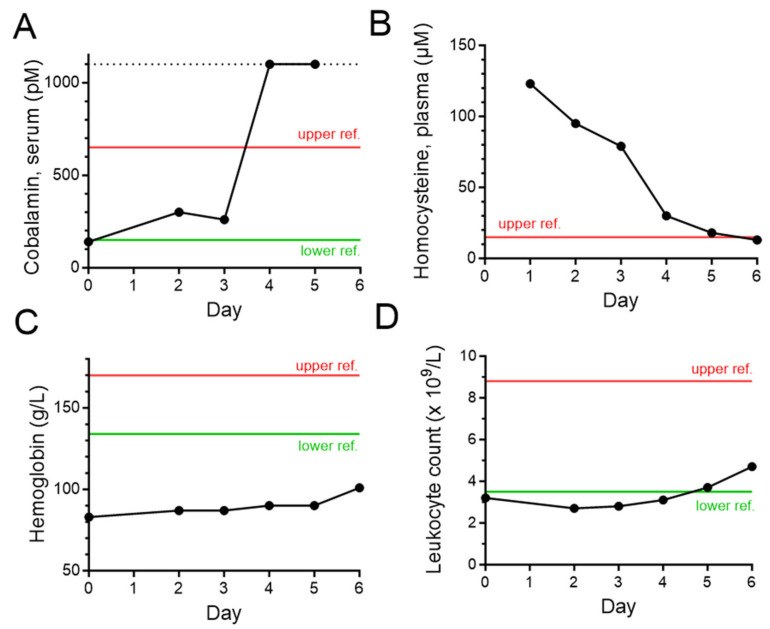
Clinical chemistry dynamics during the first week of admission. Day 0 represents the arrival to the emergency room and the time course (black) shows the evolution during the initial hospitalization. Green and red lines represent lower and upper reference intervals. The dotted line represents the maximum analysed concentration. (**A**) Serum cobalamin analysis does not differentiate between functional cobalamin and oxidized cobalamin. Thus, the value of 140 pmol/L likely underestimates the true level of cobalamin deficiency on presentation. (**B**) Plasma homocysteine was 123 µmol/L on day 1, indicating a severe impairment of the methionine cycle. (**C**) The patient was anaemic on presentation, with haemoglobin of 83 g/L. The anaemia was microcytic (mean corpuscular volume, 71 fL; reference, 82–98 fL) and ferritin was low (3 µg/L; reference, 30–400 µg/L), suggesting iron deficiency. The patient was treated with oral iron sulphate tablets. It is possible that bone marrow dysfunction caused by N_2_O contributed to the anaemia (see (**D**)). (**D**) The leukocyte count was low on arrival and normalized in 5 days. This may have been due to bone marrow dysfunction caused by N_2_O. The patient had a normal thrombocyte count.

Laboratory testing revealed low serum cobalamin and high plasma homocysteine levels (see Figure 1). The patient was treated with subcutaneous hydroxocobalamin injections, 2 mg twice daily for 4 days, and was thereafter switched to oral cyanocobalamin, 1 mg per day.

**Figure 2:**
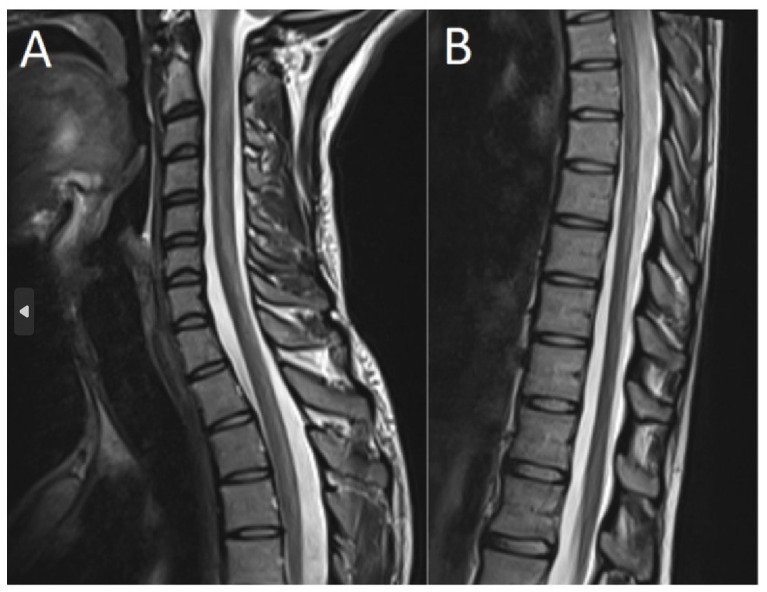
T2-weighted contrast-enhanced spine MRI shows subacute combined degeneration. Contrast enhancement was observed in the cervical (**A**) and thoracic (**B**) spinal cord. Investigations were performed two days after admission.

An MRI of the spine revealed intramedullary dorsal column signal hyperintensities stretching from C2 to Th11, consistent with demyelination (see Figure 2). Brain MRI showed hyperintensities in the third and fifth cranial nerves, but these abnormalities did not have any clinical correlates. Neurophysiological tests were not performed in this case. The patient was treated for 20 days at a spinal injury ward and recovered partially with regards to motor and cognitive symptoms. At discharge, the MoCA was 26/30 points; he was able to function independently and could walk shorter distances without support. Unfortunately, the notes from the rehabilitation unit are very sparse; the patient failed to show up at scheduled follow-up visits and could not be reached by telephone or mail despite multiple attempts to contact him.

## 3. Discussion

We report a case of severe N_2_O abuse with cobalamin neuropathy including cognitive and hyperkinetic symptoms. The symptoms of cobalamin neuropathy are caused by the impaired function of two metabolic pathways that use the vitamin as a cofactor: (A) the methionine synthase pathway that turns homocysteine into methionine through methylation and (B) the methylmalonyl-CoA mutase pathway that converts methylmalonyl-CoA to succinyl-CoA [14]. Their disruption in cobalamin deficiency leads to the accumulation in the blood of homocysteine and methyl malonate, respectively. In Sweden, serum homocysteine is a widely available routine analysis with a laboratory turnaround time measured in hours, whereas serum methyl malonate is inaccessible to a degree that makes it impossible to use to guide clinical care in the acute setting.

**Figure 3:**
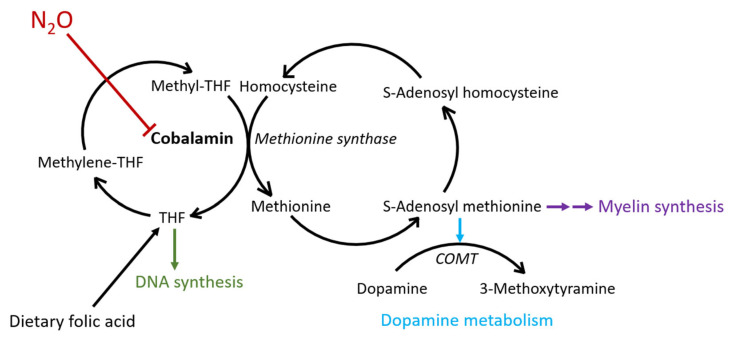
N_2_O disrupts methylation. Schematic of key steps in the methionine cycle. N_2_O (red) inactivates cobalamin that is required for the generation of the methyl donor S-Adenosyl methionine (SAM). SAM-mediated methylation is necessary for various cellular processes, including myelin sheath synthesis and dopamine metabolism via catechol-*O*-methyltransferase (COMT). When cobalamin deficiency occurs, the methionine cycle is halted, homocysteine accumulates, and SAM levels decrease. The methionine cycle is also required for the demethylation of methyl-tetrahydrofolate (THF) into THF. THF is required for normal DNA synthesis and cobalamin deficiency indirectly impairs DNA synthesis, which may manifest as macrocytic anaemia and glossitis. However, adequate dietary folate supplementation likely upholds DNA synthesis in most cases of cobalamin deficiency in N_2_O abuse (see text).

While an elevated serum homocysteine level is not specific for cobalamin deficiency; in the present case, the recent history of binge N_2_O abuse, the rapidly reversible hyperhomocysteinemia, and the typical findings of SCD on spinal MRI were sufficient to establish the diagnosis of N_2_O-induced cobalamin neuropathy without the use of serum methyl malonate. The normalization of homocysteine within a few days of N_2_O abstinence and cobalamin substitution seen in the present case is typical in the experience of the PC and indirectly suggests a return of methionine cycle function (see Figure 1 and Figure 3) [25].

The methionine cycle is necessary for maintaining an adequate cellular supply of S-Adenosylmethionine (SAM). SAM is an activated carrier molecule, structurally similar to other important carrier molecules such as ATP and acetyl-CoA, that functions as a universal methyl group donor [26]. SAM is involved in the synthesis and maintenance of myelin sheaths and depletion of SAM levels is likely to be an important cause of the demyelinating injury seen in cobalamin neuropathy [13]. However, while the return of function of the methionine cycle seems to occur rapidly after cobalamin repletion, the recovery from extensive demyelination is a much slower process. The dissociation between the apparent recovery of methionine cycle function and symptom resolution is illustrated by the persistent symptoms of spinal cord neuropathy and cognitive dysfunction long after serum homocysteine normalization in the present case.

The patient in the present case displayed symptoms associated with a hyperdopaminergic state, including dystonic posturing and athetosis, symptoms that have also been reported in a limited number of prior patients with cobalamin neuropathy [15,27]. Since one pathway of dopamine degradation is via the SAM-dependent enzyme catechol-*O*-methyltransferase (COMT), we hypothesize that N_2_O-induced hyperkinetic symptoms may be caused by COMT dysfunction secondary to depleted levels of SAM (see Figure 3) [28].

In contrast to the slow recovery from demyelination associated with SAM depletion, the recovery of COMT function and the normalization of dopamine levels are expected to be coincidental with the recovery of methionine cycle functioning. In the present case, the hyperkinetic symptoms did indeed disappear during the first days of hospitalization as serum homocysteine levels normalized (see Figure 1). Intriguingly, a recent Swedish publication describes two patients with histories of intense N_2_O abuse who were admitted to hospital with severe psychotic symptoms and hyperkinetic movements. Psychotic symptoms and the movement disorder both disappeared within days after hospitalization, which raises the possibility that psychotic symptoms in cobalamin deficiency may also be related to dopamine excess caused by COMT dysfunction [29].

The patient in our case was anaemic and had a low white blood cell count (see Figure 1). It is possible that cobalamin deficiency contributed to these symptoms. The dysfunction of the methionine cycle leads to the accumulation of folate in the form of methyl-THF which cannot be demethylated (see Figure 3). This “methyl trap” leads to a state of folate pseudodeficiency which impairs DNA and RNA synthesis [11,14]. However, unlike almost all other causes of cobalamin deficiency, including achlorhydria—the most common pathology underlying pernicious anaemia—no malabsorption is involved in cobalamin neuropathy caused by N_2_O abuse [15]. This suggests that dietary folate is absorbed normally and may bypass the “methyl trap”. This is a likely explanation for why megaloblastic anaemia and glossitis, symptoms of impaired DNA synthesis in pernicious anaemia, are usually either absent (glossitis) or mild in comparison with symptoms of neuropathy (possibly illustrated by the blood count in the present case) in cobalamin neuropathy caused by N_2_O abuse [11,14].

Many treatment protocols for cobalamin neuropathy caused by N_2_O abuse are identical to those used in pernicious anaemia, with iterated injections of hydroxocobalamin that continue for weeks to months after presentation [30,31,32,33]. However, in the absence of malabsorption, this is likely to be unnecessary. After rapidly raising cobalamin to supra-normal levels via adequate initial injections of hydroxocobalamin, further supplementation through the oral route should be sufficient. For the current patient, serial laboratory investigations confirmed the restoration of cobalamin levels, while the swift normalization of serum homocysteine served as an indicator of normalized methionine cycle function (see Figure 1). In this case, hydroxocobalamin was erroneously given subcutaneously, while it is the experience of the PC that a single intramuscular injection of 2 mg of hydroxocobalamin leads to cobalamin levels above the upper level of laboratory quantification.

The patient in the present case reported consumption of extreme amounts of N_2_O in the weeks leading up to hospitalization. This level of consumption requires easy access to N_2_O, which may explain why most of the early case reports involved dentists [8,15,34]. A European Union directive in 2014 removed the possibility to use pharmaceutical legislation to curb the trade of N_2_O aimed for the recreational market within the EU, and from around that time, the legal imports of nitrous oxide to Sweden have increased twenty-fold and are now approaching 100 tonnes/month—enough to supply the entire Swedish population with a monthly “whippit” bulb [22,35]. An additional market development is the replacement of the small bulbs (8 g N_2_O) with much larger gas cylinders [1]. There is virtually no demand for large cylinders from any legitimate businesses in Sweden (Association of Swedish bakers and confectioners, personal communication). Moreover, a single 615 g cylinder can ”whip” 40 litres of cream, or five times the average yearly per capita cream consumption, making households unlikely as a legitimate target market [36].

During peak use, the patient consumed six 615 g cylinders, or 1860 L of N_2_O, daily. Assuming a minute ventilation of 6 L/min, this suggests he was exposed to the equivalent of 20% N_2_O for the entire two weeks leading up to symptom deterioration. This level of exposure almost perfectly parallels the N_2_O exposure levels used to induce severe cobalamin neuropathy in animal models of SCD [13]. While there are earlier reports describing the use of hundreds of “whippit” bulbs daily, there can be little doubt that a market providing easy access to larger N_2_O cylinders greatly facilitates the consumption of such large volumes [30,32]. From an availability perspective, one might say “today we are all dentists”.

The long-term outcome of N_2_O abuse remains uncertain. Historical experience from pernicious anaemia suggests that up to 50% of patients with neurological symptoms suffer some degree of residual disability, and in cases with symptoms matching the severity of our case, major persistent neurological dysfunction was common [17,37]. Whether the same holds true for N_2_O-induced cobalamin neuropathy remains to be determined. A noticeable difference between the cobalamin neuropathy caused by pernicious anaemia versus that caused by N_2_O abuse is the patient age; whereas the former rarely develops before the seventh decade of life, the latter almost always befalls people under the age of twenty-five [22]. The presented case underscores that the current state of ubiquitous N_2_O availability is a public health concern.

## Data Availability

The data presented in this study are available in the article.
